# Predicting Lung Deposition of Extrafine Inhaled Corticosteroid-Containing Fixed Combinations in Patients with Chronic Obstructive Pulmonary Disease Using Functional Respiratory Imaging: An *In Silico* Study

**DOI:** 10.1089/jamp.2020.1601

**Published:** 2021-06-14

**Authors:** Omar S. Usmani, Benjamin Mignot, Irvin Kendall, Roberta De Maria, Daniela Cocconi, George Georges, Nicola Scichilone

**Affiliations:** ^1^ Airway Disease Section, National Heart and Lung Institute, Imperial College London, Royal Brompton Hospital, London, United Kingdom.; ^2^ FluidDA nv, Kontich, Belgium.; ^3^ Chemistry Manufacturing and Controls, Chiesi Farmaceutici SpA, Parma, Italy.; ^4^ Global Clinical Development, Chiesi Farmaceutici SpA, Parma, Italy.; ^5^ Division of Respiratory Diseases, Department of Promoting Health, Maternal-Infant Excellence and Internal and Specialized Medicine (Promise), G. D'Alessandro, University of Palermo, Palermo, Italy.

**Keywords:** combination drug, extrafine, functional respiratory imaging, inhaled corticosteroid, lung deposition, pressurized metered-dose inhaler

## Abstract

***Background:*** Functional respiratory imaging (FRI) is a computational fluid dynamics-based technique using three-dimensional models of human lungs and formulation profiles to simulate aerosol deposition.

***Methods:*** FRI was used to evaluate lung deposition of extrafine beclomethasone dipropionate (BDP)/formoterol fumarate (FF)/glycopyrronium bromide (GB) and extrafine BDP/FF delivered through pressurized metered dose inhalers and to compare results with reference gamma scintigraphy data. FRI combined high-resolution computed tomography scans of 20 patients with moderate-to-severe chronic obstructive pulmonary disease (mean forced expiratory volume in 1 second 42% predicted) with *in silico* computational flow simulations, and incorporated drug delivery parameters to calculate aerosol airway deposition. Inhalation was simulated using profiles obtained from real-life measurements.

***Results:*** Total lung deposition (proportion deposited in intrathoracic region) was similarly high for both products, with mean ± standard deviation (SD) values of 31.0% ± 5.7% and 28.1% ± 5.2% (relative to nominal dose) for BDP/FF/GB and BDP/FF, respectively. Pairwise comparison of the deposition of BDP and FF gave a mean intrathoracic BDP/FF/GB:BDP/FF deposition ratio of 1.10 (*p* = 0.0405). Mean intrathoracic, central and peripheral deposition ratios for BDP were 1.09 (95% confidence interval [CI]: 1.05–1.14), 0.92 (95% CI: 0.89–0.96), and 1.20 (95% CI: 1.15–1.26), respectively, and for FF were 1.11 (95% CI: 1.07–1.15), 0.94 (95% CI: 0.91–0.98), and 1.21 (95% CI: 1.15–1.27), within the bioequivalence range (0.80–1.25) for intrathoracic and central regions, and slightly exceeding the upper boundary in the peripheral region. Mean ± SD central:peripheral deposition (C:P) was 0.48 ± 0.13 for BDP/FF/GB and 0.62 ± 0.17 for BDP/FF, indicating a higher proportion of drug deposition in the small airways than in the large airways.

***Conclusion:*** FRI demonstrated similar deposition patterns for extrafine BDP/FF/GB and BDP/FF, with both having a high lung deposition. Moreover, the deposition patterns of BDP and FF were similar in both products. Furthermore, the C:P ratios of both products indicated a high peripheral deposition, supporting small airway targeting and delivery of these two extrafine fixed combinations, with a small difference in ratios potentially due to mass median aerodynamic diameters.

## Introduction

Inhaled drug lung deposition is conventionally assessed using *in vivo* scintigraphy.^(1)^ However, this involves complex methodology with product radiolabeling, and consequent exposure of patients to radiation during the procedure. Various alternatives use mathematical modeling to predict drug delivery and airways deposition.^(2)^ Functional respiratory imaging (FRI) is such a technology, combining three-dimensional (3D) lung models (obtained from standard high-resolution computed tomography [HRCT] scans) with computational fluid dynamics (CFD). In contrast to scintigraphy, FRI allows modeling of patient-specific deposition in all peripheral airways without the need for patient recruitment, and does not necessarily result in the exposure of individuals to additional radiation since data from prior studies can be reused. This technique has been validated by De Backer et al. in a crossover study evaluating regional lung deposition by FRI and single-photon emission computed tomography (SPECT) in patients with asthma, in which there was excellent agreement between calculated FRI and measured SPECT.^(3)^ Subsequent studies have confirmed the consistency between FRI and scintigraphy (Table 1).^(4–16)^

**Table 1.** Comparison between Functional Respiratory Imaging and Scintigraphy

Product	FRI (% LD)	Scintigraphy (% LD)
BDP/FF through pMDI (solution) in COPD	28^(4)^	31–34^(7,8)^
FP/FF through pMDI (suspension) in asthma	42^(9)^	41^(5)^
BUD/FF through DPI in asthma	23^(9)^	22^(10)^
BDP through pMDI (solution) in asthma	54^*^	53^(11)^

^*^ Data on file.

BDP, beclomethasone dipropionate; BUD, budesonide; COPD, chronic obstructive pulmonary disease; DPI, dry powder inhaler; FP, fluticasone propionate; FF, formoterol fumarate; FRI, functional respiratory imaging; LD, lung deposition; pMDI, pressurized metered-dose inhaler.

An extrafine formulation of a fixed-dose dual combination of the inhaled corticosteroid (ICS) beclomethasone dipropionate (BDP) and the long-acting β_2_-agonist formoterol fumarate (FF) has been available since 2006 for regular treatment of asthma, and since 2014 for symptomatic treatment of severe chronic obstructive pulmonary disease (COPD) as a pressurized metered-dose inhalation (pMDI; Foster^®^; Chiesi Farmaceutici SpA, Parma, Italy). In 2017, a fixed-dose triple combination of BDP, FF, and the long-acting muscarinic antagonist glycopyrronium bromide (GB; Trimbow^®^; Chiesi Farmaceutici SpA), in a similar extrafine pMDI formulation was approved for the maintenance treatment of COPD and is in development for severe asthma. Extrafine inhaled drugs are defined as those with particles having a mass median aerodynamic diameter (MMAD) <2 μm.^(17)^ Inhaled drugs penetrate more deeply into the smaller peripheral airways as a consequence of the particle size, with greater deposition of smaller, extrafine particles than nonextrafine.^(18)^ This may be a more optimal drug delivery pattern for inhaled medications, which would be beneficial in asthma and COPD since the peripheral airways are an important site of inflammation in both diseases.^(19,20)^ Consequently, in the development of new therapeutics a key attribute is the potential to effectively target the small airways; this ability should be assessed through the use of discriminating assessment techniques.^(21)^

In a previous study, De Backer et al. assessed lung deposition of BDP/FF (100/6 μg) in patients with severe COPD using scintigraphy.^(7)^ The average lung deposition of BDP/FF was 33.01 ± 8.9% (relative to nominal dose). From *in vitro* studies, the MMAD of BDP/FF/GB and BDP/FF is similar (BDP/FF/GB 1.1 μm; BDP/FF 1.3 μm). However, even small differences in MMAD influence deposition,^(22)^ and it was therefore important to formally evaluate BDP/FF/GB deposition. Given the good consistency between FRI and scintigraphy,^(4–16)^ we decided to use FRI for this evaluation.

## Materials and Methods

FRI methodology is based on four building blocks: (1) patient-specific 3D airway geometry modeling; (2) inhaler characteristics; (3) inhalation profile; and (4) CFD simulations to model lung deposition.

### Patients and 3D airway modeling

**FIG. 1.**
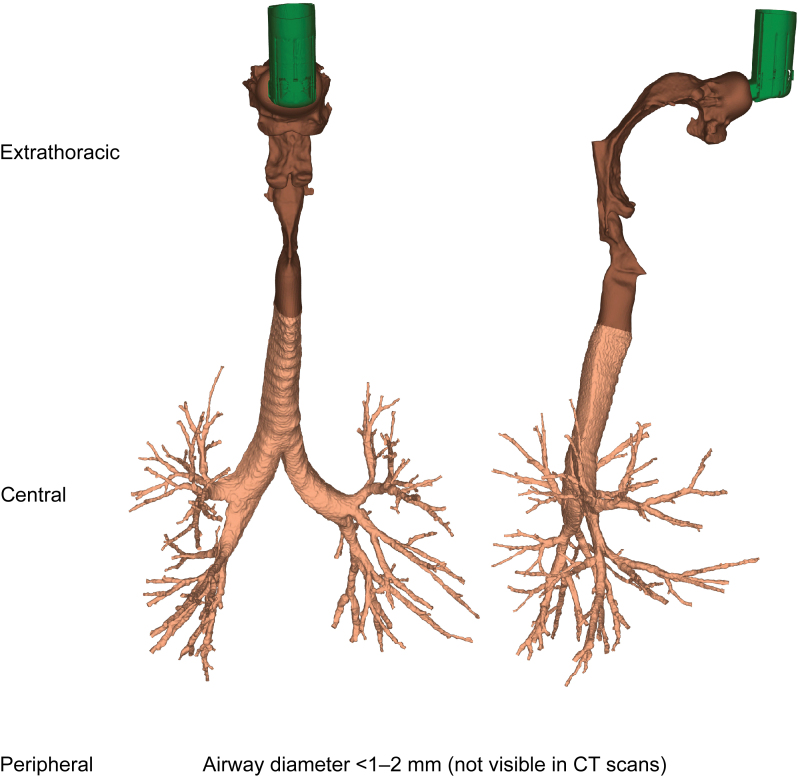
Patient-specific three-dimensional model of the extrathoracic and intrathoracic airways generated by HRCT scans. CT, computed tomography; HRCT, high-resolution computed tomography. Color images are available online.

This study investigated respiratory aerosol drug delivery in 20 patients with COPD and moderate-to-severe airflow obstruction. Patient-specific volumetric, HRCT-based 3D lung models were used, providing insights on the structural and functional characteristics of the respiratory system of each patient. HRCT scans were acquired retrospectively; informed consent was obtained from each patient, with ethics approval by the Ethics Committee of Antwerp University Hospital, Belgium. For each patient a scan was taken during inspiration and during expiration. The inspiratory scan was used to segment and model patient-specific extrathoracic (upper) and intrathoracic (lower) airways until the intraluminal and alveolar airways could no longer be distinguished—that is, airways with a minimum diameter of 1–2 mm, corresponding to a HRCT scan voxel size of ∼0.5 mm^3^. Insights on airways further downstream in the peripheral region were retrieved through the lobar expansion induced by internal airflow distribution, by using expiratory and inspiratory scans to measure the change in lobar volume from expiration to inspiration. An example of a 3D airway model from a representative patient with COPD is shown in Figure 1, illustrating the extrathoracic region (mouth and throat), the central (large) airways, and the peripheral (small) airways of the respiratory tract. Inspiratory (total lung volume) and expiratory (functional residual capacity) scans were used to produce the lung lobe data (with lobar expansion assumed to be uniform), with scans taken at the end of inhalation/exhalation.

Commercially available validated software packages (Mimics 15.0 and 3-Matic 7.0; Materialise nv, Leuven, Belgium) were used for all segmentation and modeling operations. HRCT scans were obtained from previous studies in which patient consent and approval from relevant Institutional Review Boards were procured; this study did not actively recruit patients.

### Inhaler characteristics

To evaluate lung deposition of BDP/FF/GB and BDP/FF, the aerosol plume characteristics plume mass, cone angle, plume ejection velocity, and duration were measured for a representative BDP/FF/GB and BDP/FF pMDI (Table 2). Plume ejection velocity was measured at 10 mm (just after actuator exit) and 100 mm (simulating the throat distance).

**Table 2.** Plume and Particle Characteristics for BDP/FF/GB and BDP/FF

	BDP/FF/GB			BDP/FF	
Mass (g)	0.073			0.058	
Angle (°)	21.24			15.61	
Velocity (m/s)	21.0 (at 10 mm)			11.4 (at 10 mm)	
	6.0 (at 100 mm)			5.1 (at 100 mm)	
Duration (ms)	229.4			169.0	
*API*	*BDP*	*FF*	*GB*	*BDP*	*FF*
MMAD (μm)	1.1	1.1	1.1	1.3	1.3
GSD	2.0	2.0	2.0	2.4	2.1
FPF (% of DD)	43	44	42	42	42
DD (μg)	87	5	11	85	5

API, active pharmaceutical ingredient; BDP, beclomethasone dipropionate; DD, delivered dose; FF, formoterol fumarate; FPF, fine particle fraction; GB, glycopyrronium bromide; GSD, geometric standard deviation; MMAD, mass median aerodynamic diameter.

Formulation characteristics were provided by Chiesi Farmaceutici SpA. Particle characteristics (MMAD, geometrical standard deviation, fine particle fraction [FPF], and delivered dose) of the compounds were measured using a Next-Generation Impactor for BDP/FF/GB and Andersen Cascade Impactor for BDP/FF (Table 2). Actual particle size measurements were used to create a particle size distribution as input for the CFD calculations.

### Inhalation profile

Two types of inhalation flow profile were tested, optimal, and measured, to observe differences in regional deposition during ideal and real-life device use, respectively. All patients were coupled to the same optimal flow profile, specifically a slow and deep inhalation with a duration of 4–5 seconds, to achieve the ideal flow rate of ∼30 L/min.^(23)^ Measured flow profiles came from the inspiratory volume and inspiratory time of 20 patients with COPD, matched by disease severity to those from whom we obtained the 3D lung models (the profiles for an example patient are shown in Supplementary Fig. S1).^(24)^ These were used to generate a realistic and representative inhalation profile for each COPD patient scan used in the study. The mean flow of the measured flow profiles was 29.35 L/min (range 16.15–68.83 L/min).

### CFD simulations

The 3D respiratory tract model was divided into two regions: extrathoracic (from mouth to extrathoracic airways) and intrathoracic (from top of the sternum to airways further downstream). This is similar to the global regions defined in scintigraphy, where the intrathoracic region is determined by the lung borders on an ^81m^Krypton ventilation scan and the extrathoracic region accounts for the upper parts of the respiratory tract, that is, the oropharynx and trachea. However, in scintigraphy, the extrathoracic region also includes the esophagus and stomach. Furthermore, for the 3D model, the intrathoracic region is subdivided into the central airways, with a diameter above 1–2 mm visible on a HRCT scan, and the peripheral airways that are not visible in the HRCT scan (Fig. 1).

Triangulated surface meshes created in 3-Matic (Materialise nv, Leuven, Belgium) were converted to tetrahedral 3D volume meshes using TGrid 14.0 (Ansys Inc., Canonsburg, PA). Subsequently, CFD simulations were performed on the 3D models by taking into account the boundary conditions: the inhalation profile is applied at the inhaler inlet to account for flow turbulence generated by the device; the percentage of flow exiting the model toward a lobe is equal to the relative lobar expansion obtained from patient-specific inspiratory and expiratory lobar 3D models; particles not deposited in the extrathoracic or central airways are considered to be deposited in the peripheral airways; no-slip conditions are chosen for the airway walls, that is, particles are trapped when hitting the wall. All inhaled particles are assumed to deposit, and so exhalation of particles cannot be modeled. The mathematical model and appropriate boundary conditions have been validated and previously published.^(3)^

### Statistical analyses

Analyses were conducted using R version 3.2.5 or higher (R Foundation for Statistical Computing, Vienna, Austria). To analyze intrathoracic BDP/FF deposition, a linear model was used. Deposition values (as percentage of nominal dose) were logarithmically transformed before analysis. The model included fixed effects for product (BDP/FF/GB and BDP/FF) and flow (optimal and measured flow). The Satterthwaite approximation for degrees of freedom was used. The mean deposition ratio (standard error) of BDP/FF/GB over BDP/FF for the intrathoracic BDP/FF deposition was obtained by pairwise comparison (significance level, *p* < 0.05) of the estimated marginal mean of intrathoracic BDP/FF deposition for each product at optimal and measured flow, respectively.

To investigate statistical equivalence between the deposition patterns of BDP/FF/GB and BDP/FF for each of the common active pharmaceutical ingredients (APIs), that is, BDP and FF, a two one-sided *t*-test (significance level, *p* < 0.05) was used to assess paired differences in deposition patterns per lung region. The equivalence plot presents the mean ratio, with 95% confidence interval (CI), of BDP/FF/GB over BDP/FF for the deposition of each common API per lung region. According to the Food and Drug Administration guidance for Industry on Statistical Approaches to Establishing Bioequivalence, the calculated confidence interval of the ratio Test over Reference should fall within the bioequivalence boundaries [0.8, 1.25].^(25)^

Descriptive results of the deposition in the global (i.e., extrathoracic and intrathoracic) and lobar regions are given as mean ± standard deviation (SD). The central-to-peripheral deposition (C:P) ratio defines the distribution of intrathoracic deposition over the larger and smaller airways by dividing deposition in the central region by deposition in the peripheral region. C:P ratios were calculated for each individual API, with these ratios averaged over the two or three APIs to give an overall value for each product.

## Results

### Patients

Imaging data for 20 patients with moderate-to-severe COPD were selected from the FluidDA database (15 males, 5 females; mean ± SD age, 64.0 ± 7.68 years; height, 168.9 ± 8.40 cm; postbronchodilator forced expiratory volume in 1 second, 42.3% ± 14.8% predicted).

### Extrathoracic deposition

At the optimal flow mean ± SD deposition in the extrathoracic region was 55.3% ± 4.8% for BDP/FF/GB and 56.7% ± 5.6% (nominal dose) for BDP/FF. At the measured flow, extrathoracic deposition was 55.1% ± 5.9% for BDP/FF/GB and 55.9% ± 5.1% (nominal dose) for BDP/FF (Supplementary Tables S1 and S2; Supplementary Fig. S2).

### Intrathoracic deposition

**FIG. 2.**
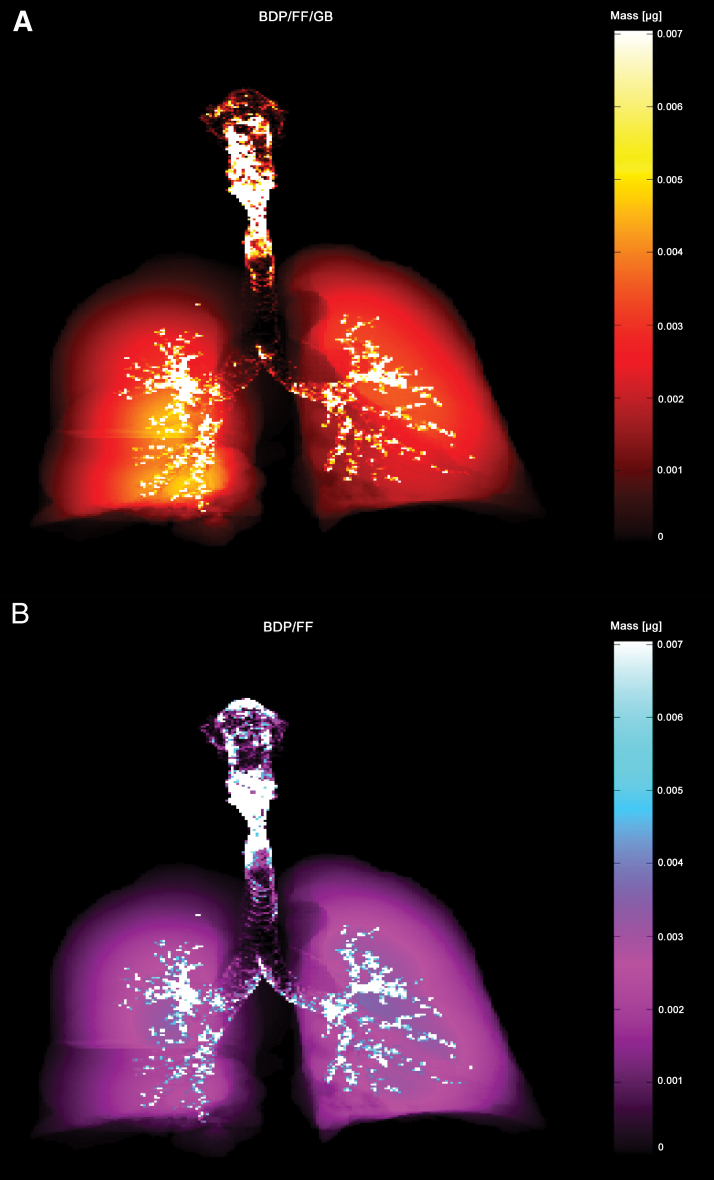
Representative modeled deposition of **(A)** BDP/FF/GB and **(B)** BDP/FF from one patient. Scintigraphy-like two-dimensional visualization in which lighter areas correspond to higher regional deposition concentrations. Both BDP/FF/GB and BDP/FF have high deposition throughout the extrathoracic and peripheral regions. BDP, beclomethasone dipropionate; FF, formoterol fumarate; GB, glycopyrronium bromide. Color images are available online.

At the optimal flow, total lung deposition (defined as the proportion of the formulation that reached the intrathoracic region) was similarly high for both products, with mean ± SD values of 30.9% ± 4.5% for BDP/FF/GB and 27.3% ± 5.6% (nominal dose) for BDP/FF. Results were similar at the measured flow, with mean ± SD values of 31.0% ± 5.7% for BDP/FF/GB and 28.1% ± 5.2% (nominal dose) for BDP/FF (Supplementary Tables S1 and S2; Supplementary Fig. S2). The deposition in the lungs and airways of an example patient is depicted in Figure 2.

### C:P ratio

At the optimal flow, the mean ± SD C:P ratio for BDP/FF/GB was 0.52 ± 0.14 and for BDP/FF was 0.65 ± 0.17. At the measured flow, the ratios were 0.48 ± 0.13 for BDP/FF/GB and 0.62 ± 0.17 for BDP/FF, indicating that higher drug deposition occurred in the small airways relative to the large airways. At the two flow profiles, BDP/FF/GB and BDP/FF had consistent central and peripheral patterns. The central and peripheral patterns of BDP/FF/GB and BDP/FF at optimal and measured flow are given in Supplementary Tables S1 and S2. Descriptive results of the deposition patterns at the measured flow are given in Supplementary Figure S2.

### Lobar deposition

The deposition (as a percentage of nominal dose) in each lung lobe was calculated at both the optimal and the measured flow for BDP/FF/GB and BDP/FF (Supplementary Tables S3 and S4). BDP/FF/GB and BDP/FF showed similar lobar deposition patterns, equally distributed over the left and right lung halves. Descriptive results of the lobar deposition patterns at the measured inhalation flow are given in Supplementary Figure S3.

### Equivalence between BDP/FF/GB and BDP/FF

For BDP and FF, as delivered by both products, the mean intrathoracic deposition (as percentage of nominal dose) ratio of BDP/FF/GB over BDP/FF was calculated at the optimal and the measured flow. The mean deposition ratio was 1.14 at the optimal flow and 1.10 at the measured flow. This indicates that at both flow profiles the intrathoracic deposition of the BDP and FF components were comparable for the BDP/FF/GB and BDP/FF pMDI products (Table 3).

**Table 3.** Mean Deposition Ratios by Pairwise Comparison of the Estimated Marginal Mean of the Intrathoracic Deposition of Extrafine BDP/FF for the Optimal and Measured Inhalation Flow Profile

Inhalation flow profile	BDP/FF/GB vs. BDP/FF ratio (SE)	*p*
Optimal	1.14 (0.052)	0.0049
Measured	1.10 (0.051)	0.0405

Significance of the statistical test is indicated by *p* < 0.05.

BDP, beclomethasone dipropionate; FF, formoterol fumarate; GB, glycopyrronium bromide; SE, standard error.

Statistical equivalence between BDP/FF/GB and BDP/FF for the deposition of BDP and FF was also calculated for the intrathoracic, central, and peripheral regions (Table 4; Supplementary Fig. S4). All mean deposition ratios and 95% CIs in the intrathoracic and central region for BDP and FF were within the range of 0.80 and 1.25. In the peripheral region the CIs slightly exceeded the upper limit of the upper bioequivalence boundary. When compared with BDP/FF, the BDP and FF particles from BDP/FF/GB had smaller MMADs but higher FPFs, with resultant higher deposition deeper in the lungs.

**Table 4.** Statistical Equivalence for the Deposition of the Common Compounds BDP and FF Per Lung Region for the Optimal and Measured Inhalation Flow Profile

Inhalation flow profile	API	Mean deposition ratio (95% CI)		
		Intrathoracic	Central	Peripheral
Optimal	BDP	1.13 (1.08–1.19)	0.97 (0.92–1.02)	1.24 (1.18–1.30)
	FF	1.15 (1.10–1.20)	0.99 (0.94–1.05)	1.25 (1.18–1.31)
Measured	BDP	1.09 (1.05–1.14)	0.92 (0.89–0.96)	1.20 (1.15–1.26)
	FF	1.11 (1.07–1.15)	0.94 (0.91–0.98)	1.21 (1.15–1.27)

API, active pharmaceutical ingredient; BDP, beclomethasone dipropionate; CI, confidence interval; FF, formoterol fumarate.

## Discussion

The current study is the first to evaluate the proportion of inhaled drug reaching the small airways (i.e., those with diameter <1 to 2 mm) for extrafine formulations of BDP/FF/GB and BDP/FF pMDIs. The findings yielded precise region-specific insights about inhaled particle deposition, providing a useful tool for the optimization of drug design (given it is an easy and quick method of evaluating regional lung deposition), and of therapeutic targeting and comparative performance. This study showed that a large fraction of inhaled extrafine BDP/FF/GB and BDP/FF were deposited in the lungs, specifically 31.0% and 28.1%, respectively. The C:P ratios in this study were 0.48 for BDP/FF/GB and 0.62 for BDP/FF, indicating that within the intrathoracic region approximately twice the amount of drug may reach the smaller peripheral airways than deposits in the larger central airways. The small difference in C:P ratios between BDP/FF/GB and BDP/FF could potentially be due to differences in MMAD; we have previously shown that C:P ratio decreases with decreasing MMAD (i.e., relative peripheral deposition increases).^(22)^

Deposition into the lung was calculated using FRI, an *in silico* technology that applies CFD calculations in combination with patient-specific airway and lung geometries to provide regional deposition metrics of aerosols and powders. FRI is able to assess the influence of various drug delivery parameters, such as inhalation flow profile, particle characteristics, device and disease population, on the aerosol deposition by investigating different delivery scenarios in a controlled environment. The patient-specific nature and core characteristics of FRI makes it an alternative to scintigraphy, providing similar results in a more time- and cost-effective manner without the need for active patient recruitment. In this study, the extrathoracic and intrathoracic deposition values for BDP/FF were very close to the deposition values found in the BDP/FF scintigraphy study by De Backer et al.^(7)^: the extrathoracic deposition was 55.9% of the nominal dose with FRI versus 55.0% with scintigraphy, whereas intrathoracic deposition was 28.1% of the nominal dose with FRI versus 33.1% with scintigraphy. Although FRI results are often consistent with scintigraphy (regardless of the molecules or devices; Table 1), a key difference between the techniques is how lung images are processed. Scintigraphy is a two-dimensional technique, in which small airways to the front and back of the central airways are included in the central compartment. In contrast, the 3D processing of FRI allows all peripheral airways to be assessed, regardless of location.

The C:P ratio obtained with FRI cannot be compared with the C:P ratio obtained from scintigraphy, due to differences in the definitions of the central and peripheral regions. In the scintigraphy study of De Backer et al. deposition was determined on a bidimensional planar image of a Krypton ventilation scan, in which rectangular regions of interest were drawn to define the central and peripheral regions.^(7)^ As a consequence, the central and peripheral region in scintigraphy comprises both intermediate/small airways and alveoli. In contrast, one of the major scientific advantages of the use of 3D models in FRI is that this enables aerosol deposition in the large and small airways to be distinguished and separately quantified. However, mucociliary clearance is not taken into account since it does not affect particle deposition. In contrast, redistribution of the particles following initial deposition could be impacted by clearance mechanisms, which may then affect the efficacy and safety of a compound.

A few limitations of this study are worth noting. First, the measured inhalation flow profile applied in the simulations was generated by the inhalation time and inhalation volume measurements in 20 representative patients from a previous study, rather than the 20 patients who provided the computed tomography (CT) scans for the 3D airway geometries. On an individual patient level, a more accurate deposition pattern might have been obtained if each CT scan was coupled with the inhalation flow profile from the same patient. Second, inhaled particles that were not trapped in the extrathoracic or central airways were assumed to be deposited in the smaller peripheral airways, rather than being exhaled.^(26,27)^ This assumption is supported by the results from the *in vivo* extrafine BDP/FF scintigraphy study by De Backer et al.,^(7)^ which showed that the exhaled fraction of the extrafine particles was on average only 3.4% of the nominal dose in patients with COPD.^(28)^

The deposition values calculated in this study are consistent with those in available literature on aerosol deposition, which showed similar lung deposition of extrafine drugs (MMAD <2 μm). Clinically it has been acknowledged that small airways (<2 mm diameter) are a major site of inflammation both in asthma and COPD,^(19,20)^ and small airways disease is present at all stage of the diseases.^(29–31)^ Therefore, it is important that ICS-containing inhaled drugs are able to reach the small airways, where their anti-inflammatory activity is most needed.

In conclusion, the results reported in this study further support FRI as a validated method to assess lung aerosol deposition, without the setup of a clinical trial and the modification of the drug for radiolabeling as required in scintigraphy. This FRI deposition study provided evidence of the lung deposition of extrafine BDP/FF/GB and confirmed the lung deposition of extrafine BDP/FF previously measured with scintigraphy.^(7)^ Moreover, similar deposition patterns, with high lung deposition, were found for BDP/FF/GB and BDP/FF. Furthermore, the C:P ratios of both products indicated a high peripheral deposition, supporting the small airway targeting and delivery of these two extrafine ICS-containing fixed combinations.

## Supplementary Material

Supplemental data

Supplemental data

Supplemental data

Supplemental data

Supplemental data

Supplemental data

Supplemental data

Supplemental data

## Notes

Reviewed by:

Gordon Prisk

Akira Isuda
