# Supplementary material for: Predicting Lung Deposition of Extrafine Inhaled Corticosteroid-Containing Fixed Combinations in Patients with Chronic Obstructive Pulmonary Disease Using Functional Respiratory Imaging: An In Silico Study
Source: J Aerosol Med Pulm Drug Deliv. 2021 Jun 14;34(3):204–11. doi: 10.1089/jamp.2020.1601 (PMC8219200; doi:10.1089/jamp.2020.1601)

**Supplementary Figure A.** The optimal flow profile with mean flow rate 30 L/min (blue) and an example of a measured flow profile with mean flow rate of 32.35 L/min derived from the real-life inspiratory volume and inhalation time measurements in a representative patient with COPD (red).


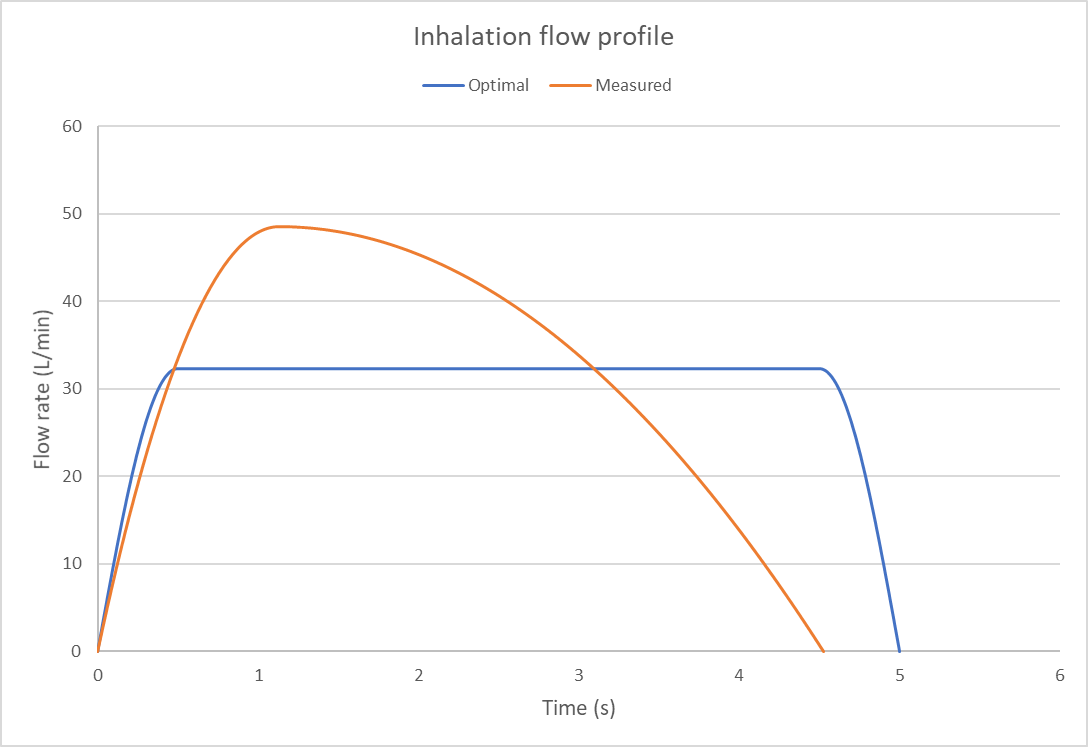

Supplement: Supplemental data [file Supp_Fig1.docx]
