# Supplementary material for: Predicting Lung Deposition of Extrafine Inhaled Corticosteroid-Containing Fixed Combinations in Patients with Chronic Obstructive Pulmonary Disease Using Functional Respiratory Imaging: An In Silico Study
Source: J Aerosol Med Pulm Drug Deliv. 2021 Jun 14;34(3):204–11. doi: 10.1089/jamp.2020.1601 (PMC8219200; doi:10.1089/jamp.2020.1601)
Supplement: Supplemental data [file Supp_Table1.docx]

# Predicting lung deposition of extrafine inhaled corticosteroid-containing fixed combinations in patients with COPD using functional respiratory imaging: An *in-silico* study

## Authors

Omar S. Usmani, Benjamin Mignot, Irvin Kendall, Roberta De Maria, Daniela Cocconi, George Georges, Nicola Scichilone

# SUPPLEMENT

**Supplementary Table A.** Modelled lung deposition (% of nominal dose) for BDP/FF/GB and BDP/FF in the global lung regions for the optimal flow profile.

| **Patient number** | **Deposition (% of nominal dose)** | | | | | | | | **Ratio (-)** | |
| --- | --- | --- | --- | --- | --- | --- | --- | --- | --- | --- |
|  | **Extrathoracic** | | **Intrathoracic** | | **Central** | | **Peripheral** | | **C:P** | |
|  | **BDP/**  **FF/GB** | **BDP/**  **FF** | **BDP/**  **FF/GB** | **BDP/**  **FF** | **BDP/**  **FF/GB** | **BDP/**  **FF** | **BDP/**  **FF/GB** | **BDP/**  **FF** | **BDP/**  **FF/GB** | **BDP/**  **FF** |
| 1 | 49.6 | 51.7 | 36.5 | 32.3 | 12.4 | 13.7 | 24.1 | 18.7 | 0.5 | 0.7 |
| 2 | 50.4 | 50.9 | 35.8 | 33.1 | 11.0 | 11.8 | 24.7 | 21.3 | 0.5 | 0.6 |
| 3 | 54.8 | 57.5 | 31.3 | 26.4 | 9.7 | 10.4 | 21.7 | 16.1 | 0.5 | 0.6 |
| 4 | 50.0 | 49.7 | 36.1 | 34.3 | 10.2 | 10.7 | 25.9 | 23.6 | 0.4 | 0.5 |
| 5 | 50.7 | 50.3 | 35.4 | 33.6 | 10.7 | 12.2 | 24.7 | 21.4 | 0.4 | 0.6 |
| 6 | 57.3 | 57.0 | 28.8 | 27.0 | 7.2 | 7.6 | 21.6 | 19.3 | 0.3 | 0.4 |
| 7 | 52.1 | 57.4 | 34.0 | 26.6 | 13.5 | 11.9 | 20.5 | 14.6 | 0.7 | 0.8 |
| 8 | 53.5 | 56.6 | 32.6 | 27.4 | 14.3 | 12.9 | 18.3 | 14.5 | 0.8 | 0.9 |
| 9 | 50.6 | 49.8 | 35.5 | 34.2 | 12.2 | 13.6 | 23.3 | 20.6 | 0.5 | 0.7 |
| 10 | 57.3 | 62.1 | 28.8 | 21.9 | 7.6 | 7.5 | 21.2 | 14.4 | 0.4 | 0.5 |
| 11 | 54.0 | 56.9 | 32.1 | 27.1 | 12.5 | 12.3 | 19.6 | 14.8 | 0.6 | 0.8 |
| 12 | 56.9 | 58.4 | 29.2 | 25.6 | 7.3 | 8.3 | 21.9 | 17.3 | 0.3 | 0.5 |
| 13 | 61.3 | 56.4 | 24.8 | 27.5 | 8.8 | 10.6 | 16.0 | 16.9 | 0.6 | 0.6 |
| 14 | 60.2 | 65.5 | 25.9 | 18.4 | 11.1 | 8.4 | 14.8 | 10.0 | 0.8 | 0.9 |
| 15 | 58.8 | 57.9 | 27.3 | 26.1 | 11.4 | 13.3 | 15.9 | 12.8 | 0.7 | 1.0 |
| 16 | 62.1 | 64.3 | 24.1 | 19.7 | 6.5 | 6.3 | 17.5 | 13.4 | 0.4 | 0.5 |
| 17 | 65.6 | 70.7 | 20.4 | 13.2 | 7.8 | 5.6 | 12.7 | 7.7 | 0.6 | 0.7 |
| 18 | 55.6 | 53.8 | 30.5 | 30.1 | 10.8 | 10.7 | 19.7 | 19.4 | 0.6 | 0.6 |
| 19 | 53.0 | 55.1 | 33.1 | 28.9 | 9.9 | 9.8 | 23.2 | 19.1 | 0.4 | 0.5 |
| 20 | 51.6 | 51.2 | 34.5 | 32.7 | 11.7 | 13.5 | 22.8 | 19.2 | 0.5 | 0.7 |
| Mean  SD | 55.3  ±4.8 | 56.7  ±5.6 | 30.8  ±4.5 | 27.3  ±5.6 | 10.3  ±2.2 | 10.6  ±2.5 | 20.5  ±3.6 | 16.8  ±4.0 | 0.5  ±0.1 | 0.7  ±0.2 |

BDP, beclomethasone dipropionate; FF, formoterol fumarate; GB, glycopyrronium bromide.
