# Supplementary material for: Predicting Lung Deposition of Extrafine Inhaled Corticosteroid-Containing Fixed Combinations in Patients with Chronic Obstructive Pulmonary Disease Using Functional Respiratory Imaging: An In Silico Study
Source: J Aerosol Med Pulm Drug Deliv. 2021 Jun 14;34(3):204–11. doi: 10.1089/jamp.2020.1601 (PMC8219200; doi:10.1089/jamp.2020.1601)
Supplement: Supplemental data [file Supp_Table2.docx]

**Supplementary Table B.** Modelled lung deposition (% of nominal dose) for BDP/FF/GB and BDP/FF in the global lung regions for the measured flow profile

| **Patient number** | **Deposition (% of nominal dose)** | | | | | | | | **Ratio (-)** | |
| --- | --- | --- | --- | --- | --- | --- | --- | --- | --- | --- |
|  | **Extrathoracic** | | **Intrathoracic** | | **Central** | | **Peripheral** | | **C:P** | |
|  | **BDP/**  **FF/GB** | **BDP/**  **FF** | **BDP/**  **FF/GB** | **BDP/**  **FF** | **BDP/**  **FF/GB** | **BDP/**  **FF** | **BDP/**  **FF/GB** | **BDP/**  **FF** | **BDP/**  **FF/GB** | **BDP/**  **FF** |
| 1 | 49.3 | 50.6 | 36.8 | 33.4 | 12.6 | 12.7 | 24.2 | 20.7 | 0.5 | 0.6 |
| 2 | 49.8 | 51.4 | 36.3 | 32.6 | 10.2 | 11.7 | 26.2 | 20.9 | 0.4 | 0.6 |
| 3 | 53.6 | 57.6 | 32.5 | 26.3 | 10.6 | 10.3 | 21.9 | 16.1 | 0.5 | 0.6 |
| 4 | 49.2 | 49.3 | 36.9 | 34.7 | 9.3 | 10.0 | 27.6 | 24.7 | 0.3 | 0.4 |
| 5 | 54.4 | 52.1 | 31.7 | 31.9 | 13.8 | 15.3 | 17.9 | 16.6 | 0.8 | 0.9 |
| 6 | 62.6 | 61.8 | 23.5 | 22.1 | 7.3 | 7.4 | 16.2 | 14.8 | 0.5 | 0.5 |
| 7 | 50.7 | 54.5 | 35.4 | 29.5 | 11.7 | 11.8 | 23.7 | 17.7 | 0.5 | 0.7 |
| 8 | 51.9 | 55.1 | 34.2 | 28.9 | 14.5 | 13.4 | 19.8 | 15.4 | 0.7 | 0.9 |
| 9 | 52.1 | 50.3 | 34.0 | 33.6 | 12.2 | 15.2 | 21.8 | 18.4 | 0.6 | 0.8 |
| 10 | 64.9 | 63.7 | 21.2 | 20.3 | 7.0 | 7.4 | 14.2 | 12.9 | 0.5 | 0.6 |
| 11 | 53.1 | 57.8 | 33.0 | 26.2 | 10.6 | 11.5 | 22.4 | 14.7 | 0.5 | 0.8 |
| 12 | 53.3 | 53.6 | 32.8 | 30.3 | 6.3 | 7.0 | 26.5 | 23.3 | 0.2 | 0.3 |
| 13 | 60.0 | 57.3 | 26.1 | 26.7 | 7.7 | 8.8 | 18.5 | 17.9 | 0.4 | 0.5 |
| 14 | 63.7 | 61.5 | 22.4 | 22.4 | 8.0 | 9.8 | 14.4 | 12.6 | 0.6 | 0.8 |
| 15 | 51.9 | 53.3 | 34.3 | 30.6 | 11.0 | 12.3 | 23.3 | 18.3 | 0.5 | 0.7 |
| 16 | 64.2 | 60.9 | 21.9 | 23.1 | 5.2 | 6.5 | 16.7 | 16.6 | 0.3 | 0.4 |
| 17 | 64.5 | 68.7 | 21.6 | 15.3 | 8.0 | 6.7 | 13.6 | 8.6 | 0.6 | 0.8 |
| 18 | 51.5 | 54.5 | 34.6 | 29.5 | 9.9 | 10.2 | 24.7 | 19.3 | 0.4 | 0.5 |
| 19 | 49.8 | 52.2 | 36.3 | 31.8 | 10.0 | 10.4 | 26.3 | 21.4 | 0.4 | 0.5 |
| 20 | 51.4 | 51.2 | 34.7 | 32.7 | 12.3 | 12.6 | 22.4 | 20.1 | 0.6 | 0.6 |
| Mean  SD | 55.1 ±5.9 | 55.9 ±5.1 | 31.0 ±5.7 | 28.1 ±5.2 | 9.9 ±2.5 | 10.6 ±2.7 | 21.1 ±4.8 | 17.5 ±3.8 | 0.5 ±0.1 | 0.6 ±0.2 |

BDP, beclomethasone dipropionate; FF, formoterol fumarate; GB, glycopyrronium bromide.
