# Supplementary material for: Predicting Lung Deposition of Extrafine Inhaled Corticosteroid-Containing Fixed Combinations in Patients with Chronic Obstructive Pulmonary Disease Using Functional Respiratory Imaging: An In Silico Study
Source: J Aerosol Med Pulm Drug Deliv. 2021 Jun 14;34(3):204–11. doi: 10.1089/jamp.2020.1601 (PMC8219200; doi:10.1089/jamp.2020.1601)
Supplement: Supplemental data [file Supp_Fig2.docx]

**Supplementary Figure B.** Deposition in the different global lung regions for BDP/FF/GB and BDP/FF for measured inhalation flow profile.


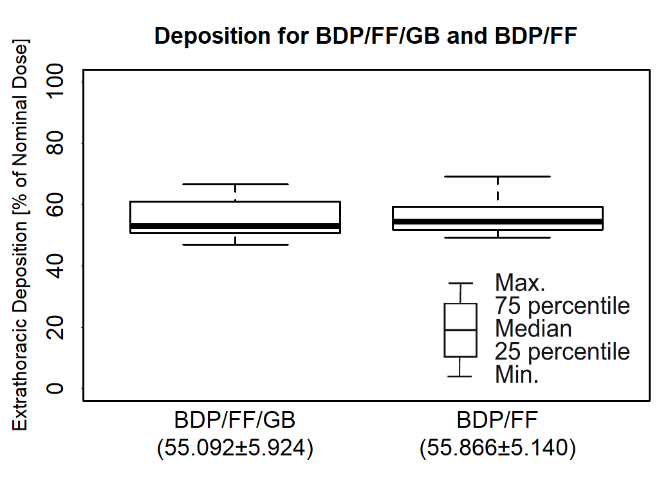









BDP, beclomethasone dipropionate; FF, formoterol fumarate; GB, glycopyrronium bromide.
