# Supplementary material for: Predicting Lung Deposition of Extrafine Inhaled Corticosteroid-Containing Fixed Combinations in Patients with Chronic Obstructive Pulmonary Disease Using Functional Respiratory Imaging: An In Silico Study
Source: J Aerosol Med Pulm Drug Deliv. 2021 Jun 14;34(3):204–11. doi: 10.1089/jamp.2020.1601 (PMC8219200; doi:10.1089/jamp.2020.1601)
Supplement: Supplemental data [file Supp_Table4.docx]

**Supplementary Table D.** Modelled lung deposition (% of nominal dose) for BDP/FF/GB and BDP/FF in the lobar lung regions for the measured flow profile

| **Patient number** | **Deposition (% of nominal dose)** | | | | | | | | | |
| --- | --- | --- | --- | --- | --- | --- | --- | --- | --- | --- |
|  | **RUL** | | **RML** | | **RLL** | | **LUL** | | **LLL** | |
|  | **BDP/**  **FF/GB** | **BDP/**  **FF** | **BDP/**  **FF/GB** | **BDP/**  **FF** | **BDP/**  **FF/GB** | **BDP/**  **FF** | **BDP/**  **FF/GB** | **BDP/**  **FF** | **BDP/**  **FF/GB** | **BDP/**  **FF** |
| 1 | 12.8 | 5.7 | 0.5 | 1.1 | 10.1 | 6.5 | 8.3 | 10.6 | 4.7 | 8.5 |
| 2 | 4.7 | 3.3 | 0.8 | 1.4 | 6.9 | 9.0 | 12.1 | 8.0 | 11.1 | 9.7 |
| 3 | 8.1 | 6.9 | 1.7 | 1.0 | 6.3 | 2.9 | 11.9 | 9.4 | 3.8 | 5.0 |
| 4 | 2.1 | 4.1 | 0.6 | 2.0 | 14.8 | 12.2 | 11.0 | 8.7 | 7.9 | 6.8 |
| 5 | 3.7 | 2.8 | 1.2 | 1.0 | 10.5 | 11.3 | 4.0 | 4.3 | 7.9 | 7.7 |
| 6 | 5.4 | 4.7 | 1.9 | 2.0 | 4.6 | 4.8 | 5.1 | 4.4 | 5.3 | 5.0 |
| 7 | 2.4 | 3.5 | 1.5 | 2.4 | 5.9 | 5.2 | 17.3 | 9.0 | 7.1 | 7.5 |
| 8 | 0.5 | 0.6 | 0.3 | 0.4 | 13.9 | 7.8 | 3.7 | 4.2 | 11.9 | 11.6 |
| 9 | 3.6 | 3.5 | 2.2 | 1.2 | 5.8 | 5.7 | 8.3 | 9.9 | 12.0 | 9.9 |
| 10 | 3.7 | 2.0 | 1.9 | 1.6 | 7.7 | 6.6 | 2.1 | 3.1 | 4.5 | 5.4 |
| 11 | 6.6 | 7.4 | 0.6 | 1.9 | 1.2 | 3.9 | 17.5 | 7.3 | 5.8 | 2.8 |
| 12 | 2.9 | 6.3 | 0.5 | 1.4 | 5.1 | 4.0 | 2.4 | 7.2 | 21.3 | 9.8 |
| 13 | 4.9 | 5.7 | 0.5 | 0.8 | 6.4 | 6.8 | 7.4 | 5.1 | 5.6 | 6.5 |
| 14 | 2.8 | 4.0 | 1.6 | 1.6 | 3.2 | 3.8 | 8.4 | 6.7 | 5.5 | 4.8 |
| 15 | 13.7 | 4.9 | 0.8 | 1.5 | 8.0 | 8.2 | 4.0 | 4.9 | 5.2 | 7.1 |
| 16 | 2.3 | 3.2 | 0.7 | 1.8 | 4.9 | 7.2 | 2.9 | 4.6 | 10.5 | 5.2 |
| 17 | 2.9 | 2.8 | 1.7 | 1.2 | 6.3 | 3.9 | 5.0 | 3.1 | 3.9 | 2.3 |
| 18 | 7.5 | 7.3 | 1.2 | 1.5 | 4.0 | 4.1 | 17.8 | 10.8 | 3.2 | 4.4 |
| 19 | 14.4 | 11.1 | 0.7 | 0.9 | 13.7 | 8.8 | 3.9 | 4.8 | 2.8 | 5.3 |
| 20 | 6.3 | 6.9 | 0.2 | 0.2 | 11.9 | 10.0 | 3.8 | 4.4 | 10.8 | 9.3 |
| Mean (SD) | 5.6  ±3.9 | 4.8  ±2.4 | 1.1  ±0.6 | 1.3  ±0.5 | 7.6  ±3.7 | 6.6  ±2.6 | 7.8  ±5.1 | 6.5  ±2.5 | 7.5  ±4.3 | 6.7  ±2.5 |

BDP, beclomethasone dipropionate; FF, formoterol fumarate; GB, glycopyrronium bromide; LLL, left lower lobe; LUL, left upper lobe; RML, right middle lobe; RLL, right lower lobe; RUL, right upper lobe.
