# Supplementary material for: Predicting Lung Deposition of Extrafine Inhaled Corticosteroid-Containing Fixed Combinations in Patients with Chronic Obstructive Pulmonary Disease Using Functional Respiratory Imaging: An In Silico Study
Source: J Aerosol Med Pulm Drug Deliv. 2021 Jun 14;34(3):204–11. doi: 10.1089/jamp.2020.1601 (PMC8219200; doi:10.1089/jamp.2020.1601)
Supplement: Supplemental data [file Supp_Fig3.docx]

**Supplementary Figure C.** Deposition in the different lung lobes for BDP/FF/GB and BDP/FF for the measured inhalation flow profile.















BDP, beclomethasone dipropionate; FF, formoterol fumarate; GB, glycopyrronium bromide.
